# Supplementary material for: Hybrid Models and Biological Model Reduction with PyDSTool
Source: PLoS Comput Biol. 2012 Aug 9;8(8):e1002628. doi: 10.1371/journal.pcbi.1002628 (PMC3415397; doi:10.1371/journal.pcbi.1002628)
Supplement: Text S4 — Complete source code for the PyDSTool package (version 0.88.120504). Includes API documentation and help files linking to web pages. This file is identical to the current public release on Sourceforge.net. (ZIP) [file pcbi.1002628.s004.zip › PyDSTool/html/PyDSTool.Toolbox.FR-module.html]

xml version="1.0" encoding="ascii"?


PyDSTool.Toolbox.FR


| Home | Trees | Indices | Help | PyDSTool |
| --- | --- | --- | --- | --- |

Package PyDSTool ::
Package Toolbox ::
Module FR

[hide private]

[frames] | no frames]

# Module FR

source code


|  |  |
| --- | --- |
| Classes | [hide private] |

vertex

edge  
v -> u directed edge

cool


|  |  |
| --- | --- |
| Functions | [hide private] |

|  |  |
| --- | --- |
| cool\_simple(t, t0, cfac) | source code |

|  |  |
| --- | --- |
| itercool(t, t0, n=30) | source code |

|  |  |
| --- | --- |
| out\_degree(V, E) | source code |

|  |  |
| --- | --- |
| in\_degree(V, E) | source code |

|  |  |
| --- | --- |
| FR(V, E, W=`[`0`,` 1`]`, L=`[`0`,` 1`]`, num\_its=30, fixed=`[``]`)  Fruchterman - Reingold graphing algorithm. | source code |


|  |  |
| --- | --- |
| Variables | [hide private] |

tarray = `array([ 0.98, 0.92, 0.86, 0.78, 0.72, 0.67, 0.6...`


|  |  |
| --- | --- |
| Function Details | [hide private] |

|  |  |
| --- | --- |
| FR(V, E, W=`[`0`,` 1`]`, L=`[`0`,` 1`]`, num\_its=30, fixed=`[``]`) | source code |

Fruchterman - Reingold graphing algorithm.

Changes V and E in-place.

  


|  |  |
| --- | --- |
| Variables Details | [hide private] |

### tarray

Value:
:   ```
    array([ 0.98,  0.92,  0.86,  0.78,  0.72,  0.67,  0.64,  0.6 ,  0.5 ,
            0.45,  0.4 ,  0.35,  0.3 ,  0.26,  0.2 ,  0.16,  0.12,  0.09,
            0.04,  0.  ])
    ```

  


| Home | Trees | Indices | Help | PyDSTool |
| --- | --- | --- | --- | --- |

|  |  |
| --- | --- |
| Generated by Epydoc 3.0.1 on Fri May 4 15:24:04 2012 | http://epydoc.sourceforge.net |
